# Gut Microbiota, Microbial Metabolites and Human Physical Performance

**DOI:** 10.3390/metabo11110716

**Published:** 2021-10-21

**Authors:** Sanna Lensu, Satu Pekkala

**Affiliations:** 1 Faculty of Psychology and Education, University of Jyväskylä, 40014 Jyväskylä, Finland; sanna.t.k.lensu@jyu.fi; 2 Faculty of Sport and Health Sciences, University of Jyväskylä, 40014 Jyväskylä, Finland

**Keywords:** gut microbiota, exercise training, human, longitudinal studies, physical activity, health

## Abstract

Trillions of microbes inhabiting the gut modulate the metabolism of the host. Cross-sectional studies have reported associations between physical performance and the gut microbiota (GM). Physical activity seems to increase GM diversity and the abundance of certain health-beneficial microbes. We reviewed the evidence from longitudinal studies on the connection between physically active lifestyle or long-term exercise interventions and the GM. We made literature searches using databases of Web of Science and PubMed Medline to collect human studies showing or not the associations between the GM and exercise. Many controversies exist in the studies. However, the longitudinal studies show that frequently, medium-intensity endurance exercise has yielded most beneficial effects on the GM, but the results vary depending on the study population and exercise protocol. In addition, the literature shows that certain microbes own the potency to increase physical activity and performance. Generally, a physically active lifestyle and exercise associate with a “healthy” GM. However, in previously sedentary subjects, the exercise-induced improvements in the GM seem to disappear unless the active lifestyle is continued. Unfortunately, several studies are not controlled for the diet. Thus, in the future, more longitudinal studies on the GM and physical performance are needed, with detailed dietary information.

## 1. Introduction

A sedentary lifestyle and obesity are increasing worldwide, and as a consequence, it is estimated that physical inactivity is now a leading cause of noncommunicable diseases [1]. One factor linked with these health risks are the gut microbiota (GM), referring to the ~100 trillion microbes inhabiting our gastrointestinal tract [2]. The GM, outnumbering human cells by ~1.3 fold [2], have a long history of symbiosis with the host. Recent advances in research have expanded the understanding of multiple parallel and bidirectional roles of the GM in regulating bodily homeostasis. For instance, the GM can modulate host metabolism and satiety as well as orchestrate many immune responses [3,4].

**Figure 1:**
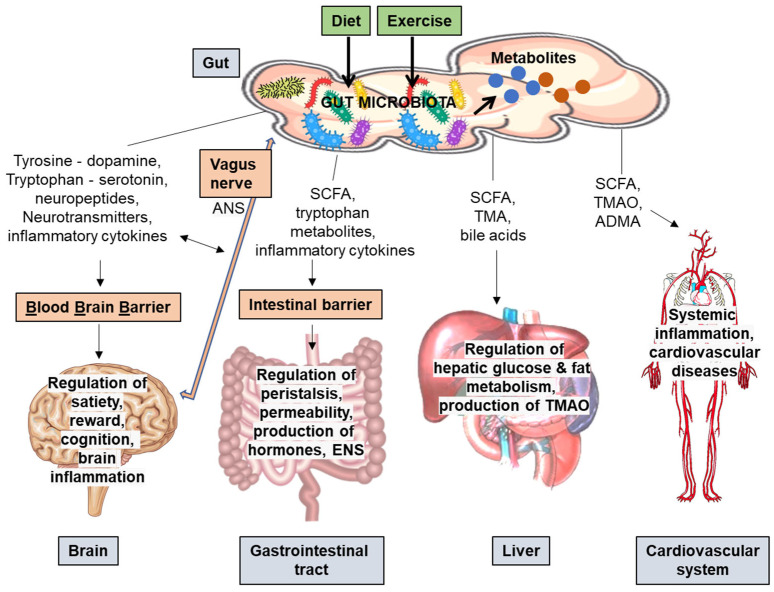
Examples of the metabolites produced by the gut microbiota (GM), and some effects of these metabolites on the bodily functions of the host. Diet and exercise are known to modulate the composition, species richness and diversity of the GM, which further leads to alterations in the production of the microbial metabolites. For example, the GM affect the metabolism of tyrosine and tryptophan that are precursors of dopamine and serotonin, respectively. Within the gut, for instance, dopamine affects the innate immune system and serotonin plays a critical role in regulating gut peristalsis. However, these gut-derived classical neurotransmitters can also have direct effects on the brain. The possible pathways include direct signaling via enteric nervous system (ENS), autonomic nervous system (ANS) and the vagus nerve, or via aryl hydrocarbon receptor-mediated pathway, or via still unknown pathways. Additionally, the GM can alter the production of several other neurotransmitters and neuropeptides. In the brain, these regulate, e.g., satiety, reward, and cognition. The GM produce short-chain fatty acids (SCFA) from the dietary fiber, for instance. These metabolites can regulate various functions in the gastrointestinal tract and affect the ENS. The SCFA can penetrate through blood brain barrier (BBB) into the brain, and have direct effects on, e.g., stimulating brain neurotrophic factors, diminishing inflammation and enhancing satiety. The SCFA together with bile acids regulate glucose and fat metabolism favorably in the liver. However, the metabolites can also be health risk factors for disease conditions. The GM-produced trimethyl amine is further converted to trimethylamineoxidase (TMAO) in the liver. TMAO has been associated with cardiovascular diseases. In addition to TMAO, the GM-produced asymmetric dimethylarginine (ADMA) is associated with cardiovascular disease outcomes under a broad range of circumstances. Higher serum ADMA may be a result of excessively metabolized proteins by the GM. In general, the GM play a critical role in regulating the innate systems, and several metabolites produced by the GM can either directly increase or decrease host inflammation. For instance, the production of SCFA can importantly reduce systemic inflammation, which improves the integrity of the gut epithelia and BBB. Abbreviations: ADMA, asymmetric dimethylarginine; ANS, autonomic nervous system; BBB, blood brain barrier; ENS, enteric nervous system; GM, gut microbiota; SCFA, short-chain fatty acids; TMA, trimethyl amine; TMAO, trimethylamineoxidase.

The effects of the GM on the physiology of the host have been recognized for a long time, and the first review articles date back to 2009 [5]. More recently, accumulating data show that it is not only the GM themselves but their production of bioactive metabolites that regulate the functions and physiology of the host (some examples in Figure 1). These molecules can act as signaling messengers by entering into the blood circulation and further, the extra-intestinal tissues [6]. For instance, the GM produce short-chain fatty acids (SCFA) mainly from dietary fiber. The amount and type of GM-produced SCFA are largely determined by the gastrointestinal transit time that in turn is affected by gut peristalsis [7], which can be remarkably modulated by physical activity [8]. In addition to the role of SCFA in modulating immune functions [9], butyrate is known to increase fat oxidation in liver [5]. As another example of the metabolites, the GM produce trimethylamine from dietary choline that is further converted into trimethylamine oxide (TMAO) in the liver. The levels of TMAO have been associated strongly with hyperglycemia, cardiovascular diseases [5] and colorectal cancer [10]. To summarize, the metabolites produced by the GM can have both positive and adverse effects on the host. Figure 1 shows examples of the GM-produced metabolites, and how they affect the bodily functions of the host.

While the diet is one of the most important determinants of the GM composition [11,12] and has been widely studied, longitudinal studies regarding the effects of exercise in shaping the GM ecosystem are scarce. Generally, the issue of the GM and exercise is affected by the type of sports and training load, in addition to genetics [13,14], gender [15], ethnicity [16], and age [17] of an athlete. The athletes usually pay special attention to their health, and thus the diet of a training athlete can be considered complete and rich in energy, nutrients, vitamins, minerals, and trace elements. However, the diet is also dependent on the type of sports, and in some sports (e.g., those with weight-based competitive classes), athletes may have strict dietary patterns and restrictions that may result in an inappropriate intake of dietary fiber and resistant starch (e.g., [18]). It has also been shown that, among athletes, the different diets at various phases of the training season affect the GM [19]. Intense exercise training also induces stress-responses mediated via hypothalamus-pituitary-adrenal (HPA) and autonomous and adrenomedullary axes that affect human physiology in various manners, including functioning of intestinal barriers and gut peristalsis, (neuro)transmitter release, and hormonal status, which have their own effects on GM as reviewed by Clark and Mach [20] based on the original papers.

In this article, we first present evidence of cross-sectional studies showing associations between the GM and physical activity. Then, we review original research articles that have determined the effects of long-term exercise on the GM. By using the term “long term exercise”, we included elite athletes or an exercise training period, which lasted for several weeks in previously inactive study participants. Studies that used dietary or other interventions in combination with exercise were excluded if there was no exercise-only group, as our intention was to review purely the effects of exercise on the GM and their metabolites. If the exercise was combined with diet intervention, the sole effects of exercise could not have been defined as diet importantly affects the GM. In Section four, we describe studies that determined the effects of the shorter exercise challenges on the GM. Finally, in Section five, we review recent findings of the effects of certain gut bacteria on physical performance.

## 2. Cross-Sectional Studies Have Reported Associations between Exercise, Physical Performance, and the Gut Microbiota in Humans—Some Examples

Numerous studies have been conducted in animals and humans to determine associations between physical activity, exercise, and the GM, and how the associations relate with different diseases or disease risk factors. These topics were not the main aim of this article, and to obtain more insight into them, several excellent review articles are available (e.g., [21,22,23,24]). The first human study showing the associations between physical activity, exercise, and the GM dates to 2014. Clarke and co-workers studied the GM of professional rugby players and compared them to two groups of sedentary individuals that were matched to the athletes by body mass index (BMI, because of the physical size of the rugby players) [25]. This cross-sectional study revealed that the athletes had higher alpha-diversity of the GM, which was positively associated with the dietary protein intake. In addition, the relative abundance of several bacterial taxa was higher in the athletes than in the controls [25]. Among others, the relative proportion of the genera *Akkermansia*, considered to be beneficial for health, was higher in the athletes. *Akkermansia* has been associated with overall good health [26] and is less abundant among obese individuals [27]. In a clinical trial, *Akkermansia* was shown to reduce overweight and improve insulin sensitivity [28]. A further metagenome analysis of the subjects from the 2014 study [25] revealed that the GM genes of the professional rugby players were enriched for pathways involved in carbohydrate metabolism, amino acid and SCFA synthesis, and the differences between the groups were larger than those in microbial composition [29]. However, the dietary intakes of total energy, protein, carbohydrates, fat, sugars, and fiber were significantly higher in the athletes than in the sedentary controls [25], which may have affected the outcomes of the GM analyses.

Another cross-sectional study also showed a positive association between *Akkermansia* and the level of physical activity. Bressa et al. compared the GM composition of sedentary or physically active women that performed at least three hours of exercise per week [30]. The study groups did not differ from each other in age, height, weight, or BMI. The GM metagenomic analysis revealed a higher abundance of Verrucomicrobia among many other taxa. Because *Akkermansia muciniphila* is the only known species of this phylum, the difference was further quantified with PCR, which confirmed that *A. muciniphila* was more abundant in the physically active than inactive women [30]. In another cross-sectional study, Verrucomicrobia was also shown to be more abundant in elderly men with lifelong training background compared with controls [31]. It is not known how a physically active lifestyle would increase the abundance of *Akkermansia*, but it could be that the dietary habits of the active individuals play a role. The physically active women [30] and the athletes in the study by Clarke et al. [25] had higher intake of dietary fiber than the sedentary counterparts, and the fiber is knowingly degraded by *Akkermansia* to produce SCFA [32]. Furthermore, the rugby players had higher intake of protein [25]. In a recent study, a high intake of protein (together with a ten-week resistance training) was shown to increase the abundance of *Akkermansia* [33]. Thus, it is likely that the diet affects the abundance of *Akkermansia*, possibly in some cases even more than the physical activity itself.

Generally, while the effects of exercise and physical activity on the GM composition appear variable, the production of the gut derived SCFA is consistently shown to increase in response to exercise [21,23,24,25]. There might be several reasons explaining the effect of exercise on this. In addition to the bacterial species within the gut and the optimal availability of substrates, one of the limiting factors for the SCFA fermentation is the intestinal transit time that additionally affects the GM composition. Increased gut motility seems to be linked with higher availability of substrates, both carbohydrates and amino acids, within the distal colon. This is due to the fast passage of substrates through the proximal parts, resulting finally in increased bacterial metabolic processes in the distal parts and increased fermentation efficiency [34]. It seems also possible that during exercise, the by-product of skeletal muscle energy metabolism, lactate, is used either as an electron sink [7] or as a carbon source for specific SCFA-producing *Veillonella* bacteria [35]. Lactate appears to be transported into the gut where it can enhance the growth of the bacteria that use it in the production of extra energy (=propionate). Propionate can improve exercise performance, and simultaneously, the bacterial breakdown of lactate prevents the accumulation of it [35].

The interplay between the GM and exercise was also shown in a cross-sectional study by Estaki et al. [36], in which physical performance could explain over 20 percent of the GM species richness in humans while, however, the dietary protein intake and age were the strongest predictors of the GM composition [36]. By studying the GM metagenomes, the authors found that physical performance associated—more than with the GM composition itself—with many GM functions including the production of butyrate and other fatty acids. The maximal oxygen uptake peak was most strongly associated with bacterial motility proteins including proteins involved in flagella assembly, and chemotaxis. These functional changes could be linked to increased intestinal peristalsis and motility by physical activity [8]. To support this view, the authors also found that cardiorespiratory fitness was associated with the production of butyrate [36], that, as mentioned above, is largely determined by the intestinal peristalsis [7].

There are also cross-sectional studies on the effects of lifelong exercise on the GM and markers of physical fitness. Recently, Šoltys et al. [31] compared the groups of training athletes and controls, who were healthy elderly men (60–70 years) in Slovakia. Šoltys and co-workers were able to show that lifelong strenuous endurance training affects the GM composition in comparison to the controls [31]. The training background did not affect the alpha-diversity of the GM, calculated as the Shannon, Simpson or Chao1 indices, but at the genus level, the trained men had higher relative abundance of e.g., *Phascolarctobacterium*, *Prevotella*, and *Subdoligranulum* than the controls, and lower abundance of *Bacteroides*, *Blautia*, *Faecaelibacterium* and *Roseburia*. The lifelong exercise was detectable as distinct differences in the selected GM genera, beta-diversity, *Bacteroides* to *Prevotella* ratio and in the physiological fitness markers. However, among the best predictors for discriminating the active ones from the controls was the *Bacteroides* to *Prevotella* ratio, in addition to maximal oxygen intake (VO_2_max) and BMI [31]. Similarly, in studies of elite top-level endurance type of athletes (males and females, aged 14–72) in Poland [37] and in Japan (females, ~20 years old) [38], distinct differences in the GM have been depicted, in comparison to the respective control population. In both studies, some markers of diversity or richness of the GM were higher among athletes than in the controls. However, in the Polish athletes the abundance of Firmicutes was higher than in the controls, equal to the previous finding [25]. It is interesting that the ratio of Firmicutes to Bacteroidetes was higher, as it has been associated with heightened disease risk and obesity [39]. However, in addition to obesity, data from animal [40] and human [41] experiments suggest that the increased ratio may also be linked to the enhanced exercise capacity, i.e., enhanced VO_2_max. Among Japanese female athletes, the abundance of *Faecalibacterium*, *Mucispirillum*, *Haemophilia* and *Rothia* was higher than in the controls. The fecal metabolites were also studied, but no difference in the concentration of butyrate was found, while the concentration of succinate was higher in the athletes than in the controls. The accumulation of succinate was suggested to cause dysbiosis within the gut and further, increased osmosis and diarrhea [38], but these conclusions warrant more research.

## 3. Longitudinal Studies Showing Effects of Longer-Term Exercise on the Human Gut Microbiota and Microbial Metabolism

In our literature search from between June and October 2021, since 2012, altogether, 1272 related publications were found, of which we have included 24 longer-term and 20 cross-sectional or short-term studies. We also included other relevant literature, making the total number of references 95. In the literature search, we included “All fields” in the searches from the National Library of Medicine, PubMed Medline. In the Web of Science, all databases were included in the search for the “Topic”. Using the keywords “gut microbiota, exercise, human” all databases of Web of Science yielded 657 publications. With the same keywords, we found 563 publications from the National Library of Medicine, in PubMed Medline search. Of these, we excluded reviews, animal experiments, sole dietary interventions, books, meeting abstracts, and duplicates. Our search with the keywords: “gut microbiota, exercise intervention, human” yielded 200 results in PubMed and 180 in Web of Science, of which eight were original articles and were the same as found with the above-mentioned search terms. Our search with the words: “gut microbiota, prolonged exercise, human” yielded 14 results on the Web of Science, of which only one was not found previously. In the PubMed search, with these keywords, all results duplicated previous search results. The screening was carried out by two independent reviewers using pre-established inclusion/exclusion criteria papers. To summarize, in our opinion, the most important longitudinal studies and their settings and findings are summarized in Table 1. The data analysis is shown in PRISMA flow diagram (Supplementary File S1). Moreover, in addition to the longitudinal studies, some review and original research publications are cited in this article, providing sources for the background information. These were considered relevant to the topic, although they were not related directly to the effects of longer-term exercise on the GM.

### 3.1. Endurance Exercise and Gut Microbiota

Allen and co-workers studied the effects of medium-intensity endurance exercise, the amount and intensity of which were progressively increased during six weeks of the intervention, on the GM of obese and normal weight study participants [42]. The exercise sessions were supervised and performed with a bicycle ergometer or on a treadmill. All participants were reported to be 100% compliant with the program, and the confounders such as diet and medication were considered. The exercise training efficiently increased lean mass and decreased body fat percentage regardless of body weight, and the relative VO_2_max increased in response to the exercise training. The authors found that 6 weeks of exercise affected the GM differently in overweight and normal weight study participants, the exercise-induced alterations in beta diversity being dependent on the obesity status. Regarding the GM composition, the endurance exercise resulted in an increase of the relative abundance of *Bacteroides* and *Collinsella* in the obese participants, while these genera decreased in the lean. *Faecalibacterium* increased in the lean participants but decreased in the obese during exercise training. The sole known species of the *Faecalibacterium* genus, *Faecalibacterium prausnitzii* is one of the most abundant commensals with important anti-inflammatory functions [61,62,63], and we have shown that this health-beneficial bacterium can ameliorate non-alcoholic fatty liver disease, too [64]. The abundance of *F. prausnitzii* has been shown to be higher in elite endurance runner women [38] and in healthy, physically active, premenopausal women than in sedentary counterparts [30], although the situation was contrary among active elderly men [31]. However, it seems that exercise may increase the abundance of *Faecalibacterium* and whether the increased abundance of this anti-inflammatory bacterium could be related to some of the anti-inflammatory effects of exercise (e.g., [65]), should be studied in the future. Because *Faecalibacterium* is known to produce SCFA, Allen et al. [42] determined the effects of exercise on the concentration of the fecal SCFA as well as on the expression of butyryl-CoA:acetate CoA-transferase (BCoAT) and methylmalonyl-CoA decarboxylase (mmdA) genes of the GM. They found that exercise increased the levels of acetate and butyrate only in the lean participants, which was accompanied with the increase in the expression of butyrate-producing BCoAT and propionate-producing mmdA [42].

After the six weeks of exercise, Allen et al. performed a “wash out period”, during which the study participants were instructed to refrain from exercise for six weeks, after which their GM were analyzed again [42]. During the wash out, the exercise-induced changes in the GM composition disappeared or were reversed to the initial level. For instance, the abundance of *Faecalibacterium* decreased in the lean participants and *Collinsella* in the obese. The levels of SCFA as well as BCoAT and mmdA gene expression decreased during the wash out period [42]. Thus, it seems that to support the exercise-induced beneficial changes in the GM, it is important to maintain the physically active lifestyle. It would also be important to determine why the GM of lean and obese individuals respond differently to exercise, and what would be the role of diet in the changes.

In our own endurance exercise study, overweight and previously sedentary women were recruited that served as controls for themselves [43]. After the six-week control period, during which the study participants were advised not to change their physical activity levels or habitual diet, they were enrolled in the six weeks endurance exercise intervention. The training sessions were supervised and performed with a bicycle ergometer, three times per week. No changes in the GM, body composition, physical performance or diet occurred during the control period. After performing the endurance exercise intervention, the dietary intakes of total energy and macronutrients were maintained indicating that the participants did not change their dietary habits. The android fat mass decreased in response to the exercise with no other changes in body composition, while the maximal power and oxygen uptake capacity increased and blood lactate levels decreased [43]. The Jaccard distance genus level beta-diversity of the GM increased in response to endurance exercise training. At the phylum level, the exercise training resulted in a decrease of Proteobacteria and an increase in Verrucomicrobia abundance. Further, the genus *Dorea*, *Anaerofilum* and *Akkermansia* increased [43]. The increase in *Akkermansia* was interesting because, as mentioned above, cross-sectional studies have shown that *Akkermansia* is more abundant in physically active individuals than in sedentary [25,30,31], and thus, *Akkermansia* might be an exercise-responsive taxon of the GM. In addition, the decrease in Proteobacteria in response to exercise is noteworthy as this phylum includes several potentially inflammatory and disease-causing taxa [66]. We further determined whether the exercise-induced changes in the bacterial taxa were dependent on the participant’s age, weight, body fat percentage, android fat percentage, total energy intake, or intake of sucrose or fiber. As a result, among all changing taxonomic abundances only Proteobacteria and Verrucomicrobia, and further *Akkermansia*, responded to exercise training independent of the above-mentioned confounding variables. Despite the increase in butyrate producing *Akkermansia* and *Dorea*, the exercise training did not increase butyrate metabolism related genes of the GM [43], which was contrary to the findings by Allen et al. [42]. We found that exercise reduced several GM metagenomic functions including those related to metabolism of fructose, mannose, alanine, and aromatic amino acid. The decrease in these genes could be due to exercise increasing the absorption of these nutrients from the small intestine [67], which would then decrease their metabolism by the colonic GM. It is known that the majority of carbohydrate-metabolizing GM inhabit the colon [68], but more studies are needed to determine how and why exercise training affects the functions of the colonic GM.

The effects of progressively increased endurance exercise training on the GM have been studied in healthy, over 60-year-old men by Taniguchi and co-workers [44]. The study showed that the diversity of the GM was negatively associated with the blood pressure of the study participants, though the exercise training did not affect the GM diversity. The exercise increased the abundance of *Oscillospira* genus, but the significant change disappeared when it was adjusted to the amount of vegetables, seaweed and rice in the diet. The authors also found that the abundance of *Clostridium difficile* decreased independent of diet. *C. difficile* is a known pathogen that can cause severe diarrhea. Though the changes in *Oscillospira* and *C. difficile* were associated with cardiometabolic variables, it is not clear how and why exercise would affect the abundance of these two taxa. The functional GM metagenomes were predicted from the 16S ribosomal gene sequences of the GM, and the analyses showed that exercise increased the genetic information processing and nucleotide metabolism of the GM. These changes could reflect the adaptations of the GM to the increased intestinal peristalsis and transit time, which are common responses to exercise [69]. Though the exact mediating mechanisms behind these exercise-induced changes are largely unknown, one of them could be intracellular transcription factor aryl hydrocarbon receptor (AhR) within the gut. Obata and co-workers have shown that in concert with the GM, AhR in the enteric neurons of the gut acts as a regulatory node between the intestinal environment and neural communication, integrating the GM with physiological responses [70]. Independently, AhR has also been linked with exercise responses [71,72] and is activated by the SCFA butyrate [73], which raises the question whether it could participate in mediating the effects of exercise on the GM.

A Danish research team studied the effects of different exercise modes in obese and overweight participants [45]. After six weeks of higher-intensity endurance training five times per week, Kern et al. [45] found that the diversity of the GM increased, while however, no associations between the diversity and VO_2_max were seen. The GM in the group of higher-intensity training started to be inter-individually more similar than in the other, lower intensity training groups. By predicting the GM metagenomic functions from the 16S ribosomal gene sequences, it was inferred that the metabolism of carbohydrates and amino acids increased. These changes were associated with the dietary intakes of carbohydrates [45], and thus it is possible that the effects of exercise on the GM were not due to exercise only, but also due to spontaneous changes in diet affecting the GM.

The effects of a decrease in endurance exercise training volume have been studied in swimmers [46]. During the competition phase the participants trained 32.4 ± 4.8 km per week. Then the training volume decreased to 19.6 ± 8.2 km per week, and further to 11.3 ± 8.1. The authors found that the alpha-diversity of the GM decreased with decreased training volume. They further demonstrated that the genera *Coprococcus* and *Faecalibacterium* associated with short-term changes in training volume—both decreased along with the training volume. The decrease in *Faecalibacterium* is interesting as it increased in response to endurance exercise in the study by Allen et al. [42].

There are also studies finding no associations between endurance exercise and the GM. Wang et al. [47] reported no effects of a 12-week moderate-intensity endurance exercise on the GM of adolescents. The research team studied 191 teenagers (12–14 years old) of which 49 had subthreshold mood symptoms, the rest being healthy. The aerobic training was running for 30 min four times weekly, while the controls had group meetings (activities were psychological education, reading, singing and group games) [47]. Although the study did not find exercise effects, the adolescents with mood symptoms had lower GM beta-diversity than the healthy ones. Moitinho-Silva et al. [48]) did not find effects of endurance or strength training on the GM of previously inactive women and men, which the authors concluded, resulting from large interindividual variability of the GM. The training lasted for six weeks, and it was, however, effective in improving or tending to improve health-related markers of the study participants [48]. To complete the study, the authors compared the GM data of previously inactive subjects to that of elite athletes of same gender and age, and despite no differences in the measures of the GM diversity, there were some minor differences in specific genera and family. Furthermore, as the abundance of *Veillonella* was previously associated with enhanced exercise performance immediately following the marathon running [35], Moitinho-Silva and co-workers quantified the amount of it by PCR, but no differences were found between the athletes and inactive participants [48]. However, these results may be confounded by the timing of fecal sampling relative to training in the athlete group. In addition, the dietary information was not available for the athlete group.

To summarize, the reported effects or lack of effects of endurance exercise on the GM vary depending on the study. Though the exercise intervention was similar in the studies by Allen et al. [42] and Munukka et al. [43], as were the overweight study groups, and the diet was taken into account, no single changing GM taxon or the function was the same. The differences between the studies were the geographic location (US vs. Finland), and both genders in the study by Allen et al. [42] and only females in the Munukka et al. [43]. Whether these could cause the discrepant results, remains to be determined. In addition, two endurance exercise studies showed either a decrease [43] or increase [45] in the carbohydrate metabolism genes of the GM. Yet, the increase may have been caused by higher dietary intake of the carbohydrates.

### 3.2. The Effects of Combined Resistance and Aerobic Exercise on the Gut Microbiota

Cronin et al. [49] studied the effects of eight-week combined resistance and aerobic training, as well as whey protein supplements on the GM of overweight study participants who were previously physically inactive [49]. Persons engaging in regular exercise commonly consume protein supplements, the consumption of which has been positively associated with a health beneficial GM composition [74]. The exercise mode used in the study by Cronin and co-workers caused only minor modifications in the GM. After eight weeks of exercise, the diversity of gut viruses decreased only in the group that exercised and was supplemented with whey protein. No changes in the gut bacteria were detected [49]. Later, Cronin and co-workers determined whether the exercise mode affected the metagenomic functions of the GM [50]. The patients in the study were overweighted and additionally, had inflammatory bowel disease. Consistent with the previous work, this study also failed to show significant effects of eight-week combined resistance and aerobic training on the gut microbiome [50].

The effects of combined resistance and aerobic training have also been studied in patients with type 2 diabetes (T2D) [51] and non-alcoholic fatty liver disease (NAFLD) [52]. Based on the traditional cultivation techniques, the study by Pasini et al. [51] indicated that six months of exercise decreased the amount of fungi and *Candida albicans* yeast in the gut of the T2D patients [51]. At the same time, the levels of zonulin (a marker reflecting intestinal leakage) decreased in the feces. In the patients with NAFLD, eight weeks of exercise increased the relative abundance of Bacteroidetes, Euarchaeota, delta and beta Proteobacteria, while Actinobacteria decreased [52]. In addition, both studies reported improved physical performance and diminished systemic inflammation in response to exercise training. Among the NAFLD patients, the scores of hepatic illnesses and among the T2D patients, the glycemic indices improved in association with physical performance. Thus, it is suggested that the effects of exercise on the GM involve various physiological adaptations throughout the regulatory systems.

A Japanese research team determined the effects of either aerobic exercise or trunk muscle resistance exercise on the GM of women over 65 years old [53]. The study was controlled for nutrition. Morita and co-workers found that the aerobic exercise increased the abundance of *Bacteroides* genus and decreased the *Clostridium* subcluster XIVa, while the resistance exercise increased the abundance of *Clostridium* cluster IX. The authors suggested that *Bacteroides* increased because aerobic exercise is known to accelerate the intestinal transit time [69] and increase the production of SCFA, which would together decrease the pH in the colon to be optimal for the growth of *Bacteroides*. However, we think that this conclusion is somewhat hindered by the fact that the abundance of known butyrate-producing *Clostridium* subcluster XIVa decreased. In general, it seems that the change in the abundance of *Bacteroides* in response to exercise or physically active lifestyle appears to be sensitive for the study set-up and the response might depend on the preceding fitness level and gender. There are equal findings of increased abundance in response to training in another study of elderly women [75] and among subjects suffering from metabolic syndrome [76]. In general, western lifestyle [77] and consumption of dietary fat [19] associate with higher *Bacteroides* abundance, while the male gender with training history associate with lower *Bacteroides* abundance [25,31,78].

A recent study in physically inactive elderly women, conducted by Zhong et al. [54], investigated the effects of eight-week combined aerobic and resistance training. The study participants were screened for their health, and only healthy subjects (n = 12) were included. They were randomized either into control (watching health-related videos twice a month) or exercising group. Each of the one-hour training sessions consisted of warm-up and cool-down, aerobic part and resistance training with a rubber band, four times per week. The diet was neither controlled nor studied. The physical performance improved in response to the training, and various measures of physical functions were associated with the abundance of Verrucomicrobia/*Akkermansia*. Although the training did not affect the alpha-diversity of the GM, the abundances of *Phascolarctobacterium* and *Mitsuokella* increased [54].

The combined strength and aerobic exercise was also studied by Quiroga and co-workers in obese pediatric patients aged between seven and 12 years [55]. After the 12-week exercise program, the metagenomic analyses of the GM revealed that the abundance of the phylum Proteobacteria decreased [55], which is in accordance with our previous study [43]. In addition, the fecal metabolomic analyses showed decreasing trends for branched chain amino acids and glucose as well as increasing trends for formate in response to combined strength and aerobic exercise. However, only the increase in xylose and decrease in galactose were significant [55]. At the same time, the training significantly inhibited the activation of peripheral blood mononuclear cell inflammasomes. Inflammasomes are multimolecular complexes of the innate immune system that act in the body in response to microbial molecules. Their activation leads to the production of various pro-inflammatory cytokines [79].

To summarize, the effects of combined resistance and aerobic exercise on the GM were different in all studies. However, similar to other studies the abundance of *Akkermansia* associated positively with physical performance and the abundance of Proteobacteria decreased in response to exercise. The characteristics of the study populations vary a lot, some being healthy and some having important metabolic diseases. Thus, it may be that the effects of combined resistance and aerobic exercise on the GM depend on the health status of an individual.

### 3.3. High Intensity Exercise or Strenuous Training and Gut Microbiota

A research group in Finland determined the effects of interval training and continuous training on the GM and inflammatory factors in the gut of participants that were insulin resistant [56]. The interval training was performed in a bicycle ergometer for two weeks (4 to 6 s sessions with 4 min breaks) and intermittent training was done at the intensity of 40 to 60 min at 60% of VO_2_max. Motiani et al. [56] found that both exercise modes decreased the inflammation in the gut as well as the ratio of Firmicutes and Bacteroidetes. The increased ratio of these phyla has been previously associated with obesity and T2D [80]. In addition, the research [56] revealed that among the previously inactive and obese (prediabetic or diabetic) men and women, the abundance of Clostridia—known to induce inflammation [81]—decreased in response to the interval training. In this study, *Faecalibacterium* increased in response to the moderate-intensity aerobic exercise [56]. Both training modes diminished systemic inflammatory markers and increased the relative abundance of Bacteroidetes phyla, which resulted in lowered ratio of Firmicutes/Bacteroidetes. Moreover, the abundance of *F. prausnitzii* tended to increase and *A. muciniphila* decrease in response to exercise [56].

In a contrary to studies conducted in obese and/or overweight participants, a study in normal weight and slightly overweight men did not find any effects of a high-intensity interval training on the diversity or composition of the GM [57]. Furthermore, the study by Rettedal et al. [57] did not detect any associations between the GM genera and physical performance.

Responses to exercise training are known to vary among humans depending on age and genotype, for example [72,82]. In the study of Liu et al., it was also found that the effects of exercise on the GM are exercise response dependent among the pre-diabetic overweight men (~40 years old) [58]. Men were subjected to progressive high-intensity interval training for twelve weeks (3 times weekly), and the 70 min training sessions consisted of combined aerobic and strength training at the level of 80–95% of maximal heart rate. The study clearly separated the subjects to exercise-responders and non-responders based on insulin sensitivity, but interestingly the adaptations of the GM were similarly divided. Some bacteria decreased only in the responders (e.g., *Alistipes putredenis*, *Alistipes shahii*, *Bacteroides xylanisolvens*), while some increased among them (*Streptococcus mitis* group, *Lachnospiraceae bacterium*). Among the bacteria with the strongest correlation with improved glucose and insulin homeostasis were *Ruminococcus gnavus*, *Alistipes shahii*, *Streptococcus mitis* group, *Eubacterium hallii* and *Escherichia coli*, the abundance of which were also differently changed in responders and non-responders. Liu et al. [58] continued the study further with the functional and metabolomic analyses of the GM, showing segregation of functional variations and pathways according to the response to exercise. The amino acid fermentation was shifted towards the production of metabolically harmful components and colonic gases in the non-responders, while in the responders towards the production of SCFA. In terms of insulin sensitivity, several other important metabolic adaptations in the GM were found to differentiate the two exercise-responsive groups. The results were confirmed by using a random forest algorithm, to screen those differences in the GM and metabolites at the baseline, which would most efficiently predict the exercise response. Those were *Bacteroides xylanisolvens*, *B. cellulosilyticus* and GABA. The predictive model was used in study a cohort of 30 prediabetic subjects, and the previous results of exercise responses were replicated in this validation cohort with almost similar discrimination index (0.880 vs. 0.747, discovery vs. validation). Finally, the authors did an animal experiment transplanting fecal microbiota from human responders and non-responders to obese, antibiotics-treated mice. The results showed that the GM from the exercise trained responders was able to induce alterations in the phenotype, glucose homeostasis, including in the physical performance [58].

Another two papers have reported the effects of a shorter term, strenuous exercise on the GM and intestinal health. The study by Karl et al. [59] showed that, high-intensity cross-ski march for four days increased the diversity of the GM of male soldiers but decreased the relative abundance of health-beneficial microbes and increased the abundance of potentially inflammatory microbes [59]. The other original research article by Keohane and co-workers reported that 33 days lasting, 5500 km long trans-oceanic rowing race increased the diversity of the GM and the abundance of butyrate-producing bacteria of the athletes [60]. In addition, increases in the GM genes responsible for the metabolism of S-adenosylmethionine, amino acids and fatty acids were seen. However, as the number of participants was only four, the study does not have great statistical power.

To summarize, the studies described above present contradictory findings, some showing effects of high-intensity exercise on the GM and others not showing such effects. The contradictions may be partly due to differing study settings. For instance, whether the study participants were healthy or not, young or elderly, men or women, will affect the final outcomes. In addition, for instance the paper by Motiani et al. did not describe how the participants managed to perform the exercise sessions, though the sessions were supervised [56]. In all described papers, the high-intensity exercise protocols differed from each other (Table 1). In addition, although the diet was monitored in all studies except in the Motiani et al. study [56], it was not standardized in any of the studies. Thus, the diet and possible dietary changes during the exercise may have influenced the GM outcomes. The methodologies differ from a study to another and for example, fecal sampling [83] and the platform used for sequencing [84] can have their own impacts on the results.

**Figure 2:**
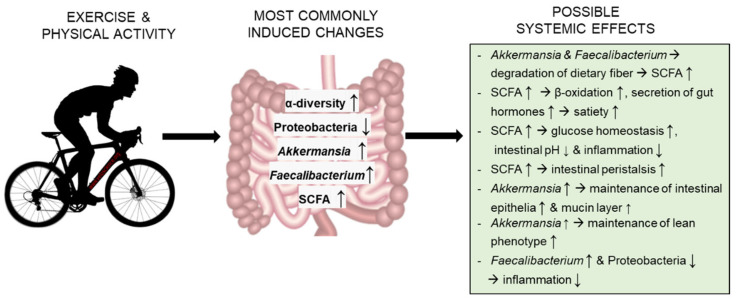
The most reproducible findings from the longitudinal exercise studies, and hypotheses of their effects on the host. Abbreviations: SCFA, short-chain fatty acids.

The Figure 2 summarizes the most common changes in the GM induced by exercise and/or physical activity, and our hypotheses on how these changes could affect the physiology of the host.

## 4. The Effects of Shorter-Term Exercise Challenges on the Gut Microbiome

Marathon is a tough endurance exercise challenge that creates great demands to the bodily functions, including the metabolism in the gut. Zhao and co-workers studied how the half-marathon affected the composition and metabolites of the GM of 20 amateur runners [85]. While the half-marathon did not affect the alpha-diversity of the GM, the linear discriminant analysis effect size (LEfSe) analysis showed that 20 taxa were enriched after running. Among these, the top five increasing genera were *Pseudobutyrivibrio*, *Coprococcus*_2, *Collinsella* and *Mitsuokella* [85]. *Mitsuokella* has been shown to also increase after eight-weeks of combined aerobic and resistance exercise [54]. The liquid chromatography/mass spectrometry analysis of the fecal metabolites revealed that the levels of 40 metabolites changed in response to the half-marathon [85]. According to Kyoto Encyclopedia of Genes and Genomes (KEGG) pathway enrichment, eight metabolites enriched into three pathways; phenylalanine, tyrosine and tryptophan biosynthesis (increased shikimic acid, decreased L-tryptophan), pyrimidine metabolism (decreased 2′3′-cyclic-uridine monophosphate, 2′-deoxyuridine-5′-diphosphate and thymine), and pentose phosphate pathway (decreased sedoheptulase-7-phosphate, increased gluconolactone and 2-deoxy-D-ribose). The family Coriobacteriaceae showed a strong correlation with the steroid, aldosterone 18-gluconide, for instance [85], which was in accordance with that Coriobacteriaceae has been shown to metabolize steroids [86]. However, Coriobacteriaceae associated positively with dietary macronutrients, and thus the availability of the nutrients during the half-marathon may have affected its abundance [85].

The GM of marathon runners was also studied by Scheiman et al. [35]. They collected fecal samples of 15 athletes who ran in the 2015 Boston Marathon, and from ten sedentary controls. Compared to the samples collected five days before marathon, in the samples five days after the marathon the genus *Veillonella* increased significantly. The authors reported that no changes were found at the phylum level and did not describe any other genus level changes in response to the marathon. Scheiman et al. [35] further studied mechanistically whether there was a causal link between the abundance of *Veillonella* and the performance of the runners. We describe the results in the next section.

Shukla and co-workers analyzed the effects of maximal exercise challenge on the GM of Myalgic encephalomyelitis/chronic fatigue syndrome (ME/CFS) patients and healthy controls [87]. ME/CFS is a disease characterized by intense and debilitating fatigue, which is not due to physical activity, and it is associated with neuro-inflammatory and oxidative processes [88]. The study participants in the work by Shukla et al. [87] were subjected to maximal bicycle ergometer test, and fecal samples were collected before the test and after 72 h [87]. The peak effort was determined based on meeting at least two of the following criteria: (1) respiratory exchange ratio ≥ 1.1, (2) achievement of 85% of age-predicted maximum heart rate, (3) rating of perceived exertion (RPE) ≥ 17, and (4) a change in VO_2_ of <200 mL with an increase in work. In the GM of the participants with ME/CFS, the relative abundance of Actinobacteria, Clostridia, Firmicutes and *Clostridium* cluster IV increased, while Proteobacteria, Bacteroidetes and *Clostridium* cluster XIVa decreased. In the healthy controls, there was an increase in Bacteroidetes, and a decrease in *Clostridium* cluster XIVa, *Clostridium* cluster IV, Proteobacteria and Firmicutes in response to exercise challenge. The authors further found an increase in the abundance of *Bacilli* in the blood samples collected from the ME/CFS patients at 48 h post-exercise, but not in the healthy controls. This could indicate that in the ME/CFS patients *Bacilli* are able to translocate from the gut to the bloodstream [87].

One study assessed the effects of three hours of medium load physical work (average 200 W/m^2^, SD ± 74) on the GM of healthy participants [89]. The activities were loading bricks, stepping, biking and arm crank at rotating intervals every 20 min in a heat chamber (34 °C, 60% relative humidity). The authors only determined the alpha-diversity of the GM as well as the relative abundance of Enterobacteriaceae and *Lactobacillus*. They observed no changes in these taxa in response to the heat and work challenge.

## 5. Certain Gut Microbes Can Increase Physical Performance

If exercise and physical activity can modify the GM of the host, based on several studies, it also seems that the GM can affect the physical activity levels or physical performance of the host. As described in the previous section, a research team at US and Canada found that the relative abundance of *Veillonella* genus increased after marathon running and thus, wanted to know whether this genus would be related to the performance of the runners [35]. They isolated and cultivated from the runner’s feces *Veillonella atypica* bacterial strain. When they intragastrically administered *V. atypica* strain to mice, the mice started to run longer distances and around 13 percent longer times compared to the control group that received normal probiotic *Lactobacillus*. Based on other omics analyses from the mice, the researchers concluded that the increased performance of the *V. atypica*-colonized mice was, at least partly, due to the exercise-induced lactate that was transported into the gut and used there by *Veillonella* to produce SCFA, which further increased the metabolism and performance of the muscle tissues [35].

Another two mechanistic studies have shown that specific microbes isolated from athlete’s feces can improve physical performance of mice. In the first study, Taiwanese researchers isolated *Bifidobacterium longum* subsp. longum OLP-01 bacterial strain from the feces of a gold medalist of the 2008 Beijing Olympics women’s 48 kg weightlifting competition [90]. Then, vehicle or OLP-01 was orally administered to mice for four weeks. The amount of OLP-01 supplementation was 2.05 × 10^9^, 4.10 × 10^9^ or 1.03 × 10^10^ colony forming units (CFU)/kg/day. The bacterium did not affect body weight or food intake of the mice. Compared to the vehicle, the OLP-01 dose-dependently increased the mean forelimb grip strengths and exhaustive swim times of the mice. In addition, after the four weeks of supplementation, the glycogen contents of the muscle and liver were higher in the mice receiving OLP-01 than in the vehicle-treated mice [90]. In another work, Lee et al. [91] isolated from the feces of the same athlete *Lactobacillus salivarius* subsp. salicinius SA-03 strain, which was also orally administered to mice for four weeks (2.05 × 10^9^, 4.10 × 10^9^, or 1.03 × 10^10^ CFU/kg/day) [91]. Compared to the vehicle, the SA-03 strain dose-dependently improved muscle strength and endurance performance of the mice, increased hepatic and muscular glycogen storage, and decreased lactate and creatine kinase levels after exercise.

After these studies in mice, the effects of OLP-01 strain administration have also been studied in humans [92]. In a double blinded, controlled trial altogether 21 volunteers received placebo or 1.5 × 10^10^ CFU/day of OLP-01 for five weeks consisting of three weeks of regular training and two weeks of de-training. Before and after the OLP-01 supplementation, the study participants were subjected to a 12 min Cooper test. The authors found that OLP-01 significantly increased the change in the 12 min Cooper’s test running distance, while it did not affect blood lactate levels. There were no significant differences in body weight, BMI, or body fat percentage between the placebo and OLP-01 groups before or after the intervention. The GM analyses showed that the supplementation decreased the relative abundance of Proteobacteria as well as increased Actinobacteria and Firmicutes. At the genus level, the OLP-01 increased the abundance of *Lactobacillus* and *Bifidobacterium* [92].

According to some studies, probiotic supplements could help to improve functioning of immune system and thus, prevent or treat the upper respiratory tract infections that are common in athletes for multiple reasons. Based on the current literature, WHO/FAO uses a following definition for probiotics: “probiotics are live microorganisms that, when administered in adequate amounts confer a health benefit on the host”. Certain probiotics are thought to decrease the numbers and activities of natural killer cells and neutrophils as well as to decrease salivary immunoglobulin A, which has importance in the infections caused by viruses and bacteria [93]. Probiotics are also suggested to provide other benefits for the performance of athletes, including absorption of certain nutrients and food items, relief against physical and mental stressors, and improvements in neurotransmitter synthesis. The effects of probiotics on exercise performance have recently been excellently reviewed by Jager et al. [94] and Calero et al. [95]. Due to this, we did not review here the effects of the probiotic bacteria on human physical performance.

## 6. Conclusions

Our review of the longitudinal studies shows that exercise affects the GM, but controversies exist. Frequently, most beneficial effects are induced by medium-intensity endurance exercise, and exercise may own potency to alleviate some diseases via GM. However, the current research has focused mainly on elderly and obese individuals as well as on athletes. Thus, more research conducted on the general population and, in both genders, is needed. The diet is a key player in terms of research on the GM, and although it is difficult to control during the long-term human studies, it should be carefully monitored. Moreover, in addition to the diet, the methodology is an important determinant of the GM results.

## Figures and Tables

**Table 1:** The main findings from the original research publications reporting effects of longer-term exercise training on the gut microbiota. Only the outcomes related to either training or the gut microbiota are shown in the table.

Training Mode	Specification and Duration of Training	Study Population	*n* of Subjects	Outcome	Reference in the List of References
ENDURANCE TRAINING					
Aerobic endurance training, increasing in duration (min) and from 60 to 75% of VO_2_max	3x per wk, 40–60 min for 6 wks, either ergometer cycling or treadmill running; post-training washout period for 6 wks	lean and obese, physically inactive women and men	*n* = 14 obese (of which *n* = 11 female), *n* = 18 lean (of which *n* = 9 female)	All subjects VO_2_max ↑, Fat % ↓ In lean *Bacteroides* ↓ *Faecalibacterium* ↑, In obese *Bacteroides* ↑, *Faecalibacterium* ↓; SCFA ↑ in feces of lean following exercise. Effects of exercise were mostly reversed during the washout. Diet was followed up and subjects were asked to maintain it; Results of diet follow-up not reported	Allen et al., 2018, [42]
Aerobic endurance training	ergometer cycling 3x per wk, for 6 wks, additional control period included for study subjects	overweighed and physically inactive women	*n* = 17 (served as own controls)	VO_2_max ↑, Android fat ↓, Proteobacteria ↓, *Akkermansia* ↑ Food records were collected throughout, diet remained mainly unaffected during exercise intervention	Munukka et al., 2018, [43]
Aerobic endurance training; cross-over	ergometer cycling 3x per wk, starting 30 min at 60% of VO_2_max level, gradually increasing in duration and intensity, for 5 wks	>60 yrs old, healthy Japanese men	*n* = 31 having both training and control periods	*Clostridium difficile* ↓, *Oscillospira* ↑ and VO_2max_ ↑ and HDL ↑, hepatic fat ↓ during exercise Of the metagenomic functions of GM, genetic information processing and metabolism of nucleotides ↑ Diet followed up with questionnaires and food records, changes in diet but they were similar during control and exercise periods	Taniguchi et al., 2018, [44]
Aerobic endurance-type, at different intensity levels	5x per wk, for 6 months; BIKE = commuting by bike, MOD, moderate exercise (50% of VO_2_ peak); VIG, vigorous exercise (70% of VO_2_peak); CON, continued habitual sedentary living	overweight or obese, inactive 20–45 yrs old women and men	BIKE, *n* = 19; MOD, *n* = 31; VIG, *n* = 24; CON, *n* = 14	Physical performance ↑ and Fat % ↓ in all exercise groups, Exercise ineffective on individual GM abundances VIG: GM α-diversity (Shannon index) ↑ after 3 months MOD: Functional capacity of GM ↑ after 3 moths Food diaries were collected, and changes occurred in exercising groups (intake of fat)	Kern et al., 2020, [45]
Endurance training (decreasing training load)	2 wk swimming 32.4 ± 4.8 km/wk, then 2 wk 19.6 ± 8.2 km/wk, then 2 wk 11.3 ± 8.1 km/wk	18–24 yrs old, collegiate swimmers	*n* = 13	GM α-diversity ↓ as well as abundance of *Coprococcus* and *Faecalibacterium* ↓ along the training volume, no changes in changes in weight, fat mass or fat-free mass	Hampton-Marcell et al., 2020 [46]
Moderate-intensity aerobic training	4x per wk, running (at level 50–70% of max. heart rate) 30 min for 12 wks	12–14 yrs old, healthy or having subthreshold mood symptoms	*n* = 49 mood syndrome and *n* = 142 healthy of which *n* = 96 were training	Exercise was ineffective No information about the diet	Wang et al., 2021, [47]
COMBINED TRAINING or STUDIES WITH VARIOUS TRAINING MODES					
Endurance/Strength/Elite athletes	Endurance: running 4x per wk >30 min (of which 1 supervised); Strength: at gym 4x per wk (of which 2 supervised). Training loads were ↑, total duration 6 wks	Inactive 20–45 yrs old, BMI 20–35 kg/m^2^, healthy women and men + elite athletes	*n* = 42 men and women → *n* = 13 endurance, *n* = 12 strength, *n* = 11 ctrl and additional group of elite athletes (*n* = 13)	Different types of exercise induced some moderate health-related effects, but no systematic effects on GM Diet changed in strength group, but other groups remained unchanged. Diet was accounted in PCA-models.	Moitinho-Silva et al., 2021, [48]
Combined resistance and aerobic training, increasing load (with or without whey supplement)	3x per wk, for 8 wks, each session included moderate aerobic training (18–32 min) + progressive, machine-based repetitive resistance exercises (starting ~70% of one-repetition max. level)	overweight and physically inactive women and men, ~35 yrs old	*n* = 25 (having exercise training only, we did not include whey groups)	VO_2_max ↑, Fat % ↓, Lean mass ↑, No significant effect on the GM α-diversity or metabolic pathways In exercise group diet remained unchanged	Cronin et al., 2018, [49]
Combined resistance and aerobic training, increasing load	3x per wk for 8 wks; session included moderate aerobic training (18–32 min) + progressive, machine-based repetitive resistance exercises (starting ~70% of one-repetition max. level)	overweight, ~25 yrs old inactive males and females diagnosed with inflammatory bowel disease (IBD)	*n* = 13 exercise group, *n* = 7 control group	In exercise group: Fat % ↓, Lean mass ↑, α-diversity of Archaea species ↑, no effects on IBD activity scores, mood or inflammation No significant effect on GM α-diversity or metabolic pathways No information about diet	Cronin et al., 2019, [50]
Combined strength/endurance/stretching training	3x per wk, 90 min per session for 6 months	type 2 diabetic males	*n* = 30	VO_2_max ↑, weight and fat-% ↓, glycemia ↑ Intestinal mycetes overgrowth ↓, zonulin (intestinal leakage) ↓, systemic inflammation ↓ No information about diet	Pasini et al., 2019, [51]
Combined strength and endurance training	3–5x per wk, for 8 wks, 10 free-weight or rubber band strength exercises and individualized endurance training (first walking/jogging then interval training)	non-alcoholic fatty liver disease patients, who completed >70% of training, age 18–70, BMI 18.5–45 kg/m^2^	*n* = 41	VO_2_max ↑, Fat % and weight ↓, hepatic illness scores ↑, inflammation ↓ Metagenomic richness ↑, Bacteroidetes and Euryarchaeota ↑, Actinobacteria ↓ Diet was not controlled/followed up	Huber et al., 2019, [52]
Trunk muscle strength or aerobic training	1 h per day for 12 wks; Aerobic = brisk walking (≥3 METs); strength = trunk muscle training (free-weight gymnastics)	healthy >60 yrs old women	*n* = 14 strength, *n* = 17 aerobic training	Both training modes: physical performance ↑, strength and elasticity ↑ Aerobic training: *Bacteroides* ↑, *Clostridium* subcluster XIVa ↓ Strength training: *Clostridium* cluster IX ↑ Diet was followed up with questionnaires and no differences during the study between groups	Morita et al., 2019, [53]
Combined aerobic and resistance training	4x per wk, ~60 min per session for 8 wks, gymnastics (= aerobic) and rubber band (= resistance) training with increase in training load	previously inactive women, ≥60 yrs old	*n* = 6 sedentary controls, *n* = 6 training	Firmicutes, *Phascolarctobacterium*, *Mitsuokella* ↑ after training, no effect on alpha diversity of GM, exercise improved physical performance No information about diet	Zhong et al., 2021 [54]
Combined strength and aerobic exercise	2x per wk, for 12 wks, bicycle ergometer, strength exercises	pediatric obese patients and non-obese, age 7–12	*n* = 39 obese → *n* = 25 exercise, *n* = 14 control, *n* = 14 non-obese	Plasma glucose ↓, Dynamic strength ↑ Proteobacteria and Gammaproteobacteria ↓ Fecal branched chain amino acids, formate, alanine and glucose ↓	Quiroga et al., 2020, [55]
HIGH-INTENSITY TRAINING					
Increasing sprint interval training or moderate-intensity aerobic training (both used cycling)	Intervals: 4–6x 30 work, 4 min rest; 3x per wk for 2 wks Endurance: 3x per wk for 2 wks, 40–60 min	obese and sedentary men and women who were diabetic or prediabetic, age 40–55 yrs	*n* = 16 (*n* = 9 type 2 diabetic, *n* = 17 prediabetic)	Interval: VO_2_max ↑, Fat %↓, Both training modes: Firmicutes/Bacteroidetes ↓, *Clostridium* ↓, *Blautia* ↓ Moderate intensity: *Faecalibacterium* ↑ No information about diet, it was asked to maintain unchanged	Motiani et al., 2020, [56]
High intensity interval ergometer training, short-term	3x per wk for 3 wks using ergometer; 8–12 bouts repeated at VO_2_max level, 1 bout = 60 s work + 75 s rest	lean and obese men, sedentary or less than 3 hrs endurance activity weekly	*n* = 14 lean, *n* = 15 obese	Insulin sensitivity and cardiovascular fitness ↑, no changes in GM, some microbes associated with insulin sensitivity among obese Diet differed between the groups before, but was not followed-up during intervention	Rettedal et al., 2020, [57]
High intensity interval training: combined strength and endurance with increasing load	3x per wk for 12 wks, supervised 70 min. sessions containing high-intensity running/biking sessions ~80–95% of VO_2_max or HR_max_ level, high-intensity resistance and calisthenics exercises (e.g., kettle ball, squats), warm-up, cooldown and stretching	non-smoking, overweight/obese men, prediabetic	*n* = 39 → *n* = 19 sedentary controls, *n* = 14 exercise responsive and *n* = 6 non-responsive, validation study with *n* = 30 obese men	Following HIIT, insulin sensitivity ↑ in responders but not in non-responders, GM α- or β-diversity unaffected, abundances of species among Firmicutes, Bacteroidetes, Proteobacteria changed in response to exercise, e.g., *Alistipes putredinis* ↓, *Bactroides xylanisolvens* ↓, *Lachnospiraceae bacterium* ↑ in responders By machine-learning algorithms GM signatures were shown to predict exercise responses in a validation study Diet was monitored with questionnaires and groups did not differ	Liu et al., 2020, [58]
STRENOUS TRAINING					
Strenous, high-intensity exercise	4 days, 51-km cross-country ski march	healthy soldiers	*n* = 73	Gut permeability ↑, GM diversity ↑ Verrucomicrobia, Tenericutes, Spirochaetes, *Lenti*sphera, *Fusobacteria* and Firmicutes ↑ Euarchaeota ↓ Fecal metabolism of phenylalanine, tryptophan and tyrosine ↓ Fecal metabolism of carbohydrates, fatty acids and secondary bile acids ↓	Karl et al., 2017 [59]
Continuous rowing	33 days, 5000 km transatlantic rowing, each athletic rowed 395 hrs	healthy, elite male athletes (~26 yrs)	*n* = 4	VO_2_max unchanged, GM α-diversity ↑, butyrate producing GM (*Roseburia hominis*, *Subdoligranulum*) ↑, *Bacteroides finegoldii* ↓; in functional metabolic pathways of GM gene products, synthesis of L-isoleucine, L-lycine ↑, S-adenosyl-L-methionine ↑, long chain fatty acids ↑, fatty acid elongation and glycolysis ↑ Diet was followed up, and macronutrient intake remained constant, but diet changed during rowing	Keohane et al., 2019, [60]

## Supplementary Materials

The following are available online at https://www.mdpi.com/article/10.3390/metabo11110716/s1, File S1: PRISMA 2020 flow diagram for new systematic reviews which included searches of databases, including published original research articles (and other sources).
