# Supplementary material for: Gut Microbiota, Microbial Metabolites and Human Physical Performance
Source: Metabolites. 2021 Oct 21;11(11):716. doi: 10.3390/metabo11110716 (PMC8619554; doi:10.3390/metabo11110716)
Supplement: Supplementary file 1 [file metabolites-11-00716-s001.zip › metabolites-1396323-supplementary.pdf]

Supplematary File S1 PRISMA 2020 flow diagram for new systematic reviews which included searches of databases, including published original research articles (and other sources)

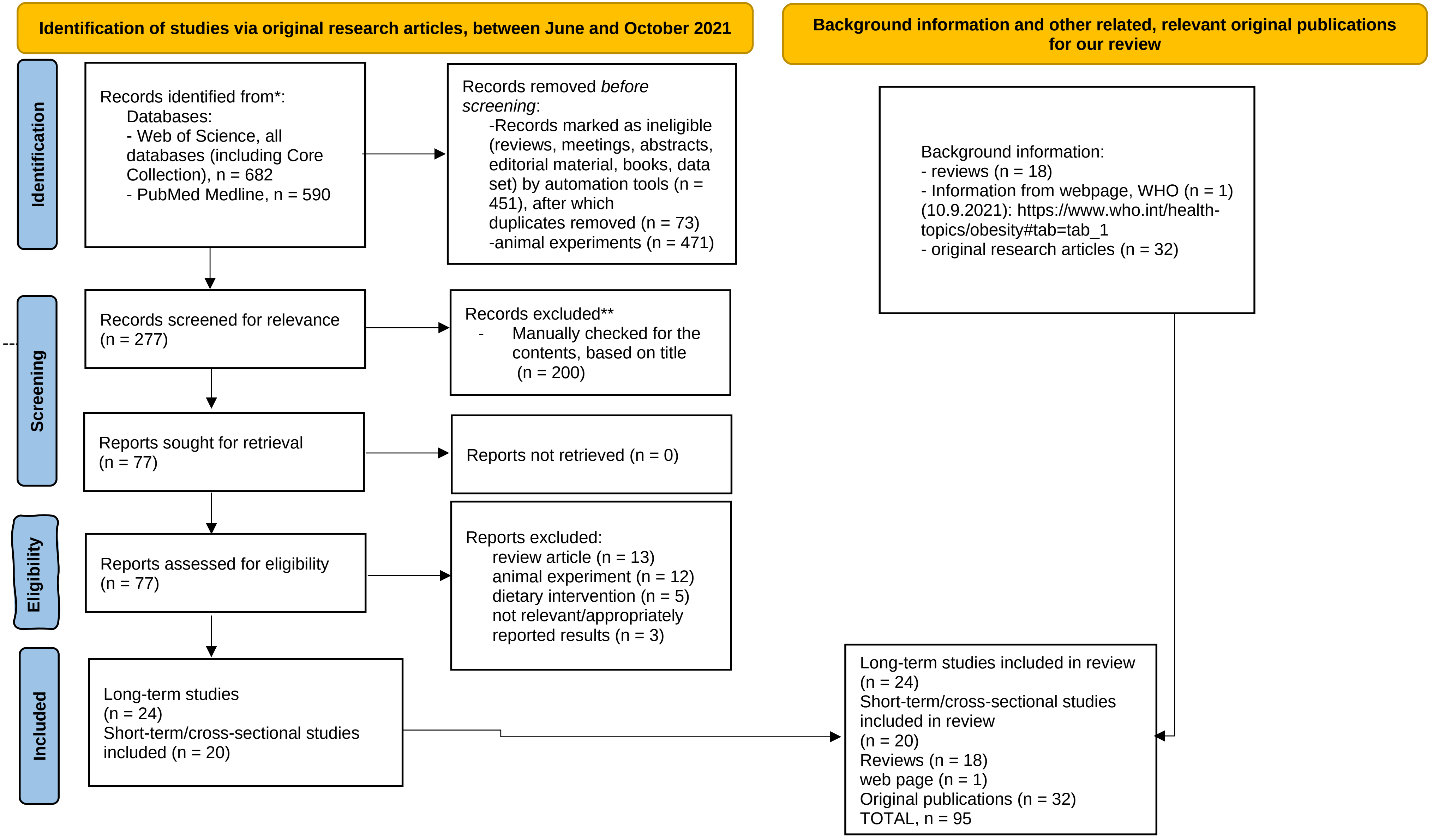

- Literature was searched for *'All fields'* in the National Library of Medicine, PubMed Medline. In the Web of Science, all databases were included in the search for the *'Topic'.* In the searches, we used the keywords *"gut microbiota, human"* in combination with exercise terms *"exercise, prolonged exercise, exercise intervention"*.

**If automation tools were used, indicate how many records were excluded by a human and how many were excluded by automation tools.

Supplematary File S1 PRISMA 2020 flow diagram for new systematic reviews which included searches of databases, including published original research articles (and other sources)

From: Page MJ, McKenzie JE, Bossuyt PM, Boutron I, Hoffmann TC, Mulrow CD, et al. The PRISMA 2020 statement: an updated guideline for reporting systematic reviews. BMJ 2021;372:n71. doi: 10.1136/bmj.n71. For more information, visit: http://www.prisma-statement.org/
